# Pleiotropic Effects on Tachyzoite and Host Cell Proteomes in Knock-Out Clones of the Open Reading Frames 297720 and 319730 Constitutively Expressed in *T. gondii* ShSp1 Tachyzoites

**DOI:** 10.3390/ijms262110433

**Published:** 2025-10-27

**Authors:** Kai Pascal Alexander Hänggeli, Joachim Müller, Manfred Heller, Anne-Christine Uldry, Sophie Braga-Lagache, David Arranz-Solís, Luis-Miguel Ortega-Mora, Andrew Hemphill

**Affiliations:** 1 Institute of Parasitology, Department of Infectious Diseases and Pathobiology, Vetsuisse Faculty, University of Bern, 3012 Bern, Switzerland; kai.haenggeli@unibe.ch (K.P.A.H.); joachim.mueller@unibe.ch (J.M.); 2 Proteomics and Mass Spectrometry Core Facility (PMSCF), Department for BioMedical Research (DBMR), University of Bern, 3012 Bern, Switzerland; manfred.heller@dbmr.unibe.ch (M.H.); anne-christine.uldry@unibe.ch (A.-C.U.); sophie.braga-lagache@unibe.ch (S.B.-L.); 3 SALUVET, Animal Health Department, Faculty of Veterinary Sciences, Complutense University of Madrid, 28040 Madrid, Spain; davidarranz@ucm.es (D.A.-S.); luis.ortega@ucm.es (L.-M.O.-M.)

**Keywords:** determinism, gene editing, host–parasite interaction, model system, systems biology

## Abstract

*Toxoplasma gondii*, the causative agent of toxoplasmosis widespread in animals and humans, is an intracellular apicomplexan protozoan parasite infecting a variety of host cells. Gene editing using CRISPR-Cas9 has become a standard tool to investigate the molecular genetics of this interaction. With respect to gene knock-out (KO) studies, the general paradigm implies that the gene of interest is expressed in the wildtype and that only the gene of interest is affected by the knock-out. Consequently, the observed phenotype depends on the presence or absence of genes of interest. To challenge this paradigm, we knocked out two open reading frames (ORFs) constitutively expressed in *T. gondii* ShSp1 tachyzoites, but not essential, namely ORF 297720 encoding a trehalose-6-phosphatase homolog and ORF 319730 encoding a You2 C2C2 zinc finger homolog. We analyzed the proteomes of tachyzoites isolated at a late stage of infection, as well as intracellular tachyzoites and host cells at an early stage of infection. The intended KO proteins were present in the *T. gondii* Sp1 wildtype but absent in the KO clones. Moreover, besides differentially expressed (DE) proteins specific to each KO, 17 DE proteins common to both KOs were identified in isolated tachyzoites and 39 in intracellular tachyzoites. Moreover, 76 common DE proteins were identified in host cells. Network and enrichment analyses showed that these proteins were functionally related to antiviral defense mechanisms. These results indicate that the KO of a gene of interest may not only affect the expression of other genes of the target organism, which in our case is *T. gondii*, but also the gene expression of its host cells. Therefore, phenotypes of KO strains may not be causally related to the KO of a given gene. Overall, this study highlights that genetic manipulation in *T. gondii* can lead to system-wide proteomic shifts in both parasite and host, emphasizing the need for cautious interpretation of knock-out-based functional analyses.

## 1. Introduction

The intracellular parasite *Toxoplasma gondii* (Apicomplexa, Alveolata, SAR [1]) infects a broad range of host cells of animal and human origin. In humans, toxoplasmosis is one of the most prevalent parasitic diseases, with one-third of the human population on Earth being chronically infected [2,3,4]. Consequently, it is not surprising that this protozoan parasite has become a major model system to study interaction with its host, with the investigations being facilitated by its amenity to genetic manipulation. In fact, for over three decades, *T. gondii* has proven its excellence as a molecular genetic model [5], and the genomes of *T. gondii* ME49 and many other strains have been sequenced (https://toxodb.org (accessed on 4 August 2025)). With respect to genetic manipulation, gene editing via CRISPR/Cas9 has become a major tool [6,7], and genome-wide screenings have allowed us to distinguish between fitness-conferring and non-conferring genes [8].

Typical CRISPR/Cas9 studies generate knock-out (KO) or knock-in (KI) strains of selected open reading frames (ORFs) with the general hypothesis: KO of ORF X is followed by effect Y. Thus, X is functionally related to Y. However, the results obtained in KO studies can only be regarded as valid if the following prerequisites are fulfilled. (i.) The genetic modification, i.e., deletion of the ORF of interest at the correct location must be demonstrated by an appropriate method. (ii.) The transcript of the ORF of interest must be present in the corresponding wildtype and absent in the KO strains. (iii). The polypeptide encoded by the ORF of interest must be present in the wildtype but absent in the KO strains. (iv.) Unspecific effects of the genetic modification procedure must be distinguished from specific effects, e.g., by comparison with unrelated KO strains in the same genetic background and/or, if available, with different clones from the same KO population. Otherwise, a correct interpretation of the phenotypes of the KO strains cannot be realized. In most studies, points i and ii are paid attention to, whereas points iii and iv are often neglected. Despite the increasing importance of whole proteome analysis, e.g., of drug-resistant vs. susceptible strains [9], such studies on KO strains are scarce. In a previously published article, we have shown that the KO of the major surface antigen SAG1 in *T. gondii* RH is correlated with pleiotropic proteome changes, notably within the cell surface proteome [10]. Thus, we hypothesize that knock-out effects extend beyond targeted genes to global proteomic changes, and may trigger secondary, system-wide effects beyond the intended target. Such pleiotropic outcomes can complicate data interpretation and limit the reliability of genotype–phenotype correlations

In this study, we compared strains with KOs in two unrelated ORFs. The first ORF is TGME49_297720 encoding a 1222 amino-acid trehalose-6-phosphatase homolog expressed in tachyzoites, bradyzoites, and oocysts (https://toxodb.org; 4 August 2025). The second of which is TGME49_319730, a 149 amino-acid YOU2 C2C2 zinc finger protein homolog located in the mitochondrion (https://toxodb.org; 4 August 2025) with unknown functions and structural similarities to the human mitochondrial inner membrane protein Tim10, identified as a major binding protein of an antiprotozoal compound [11]. We investigated whether knock-out of these two ORFs leads to broader proteomic alterations in both parasite and host cells.

## 2. Results

### 2.1. Molecular Genetic Characterization of the Knock-Outs

**Figure 1:**
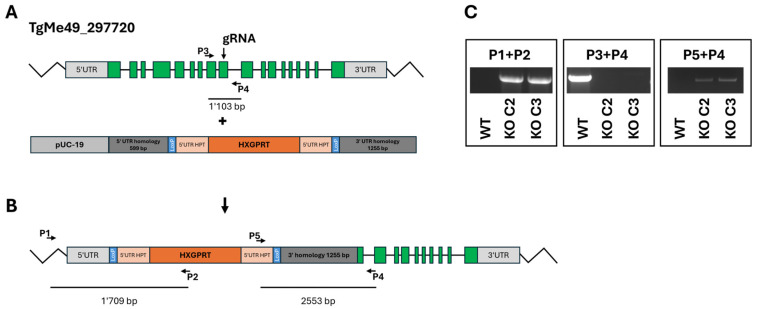
Disruption of the ORF TgME49_297720. (**A**), ORF and insert; (**B**), final construct; (**C**), quality control PCRs. The localization of guide RNA and primers P1–P5 is indicated.

The disruption (KO) of the ORF TgME49_297720 was intended to be obtained by double homologous recombination (Figure 1A) replacing the coding sequence with a repair template containing the hypoxanthine-guanine-phosphoribosyltransferase (HXGPRT) resistance marker (Figure 1B) in a ΔHPT background. However, PCR analysis revealed that the HXGPRT resistance cassette was inserted at the 5‘utr gRNA cutting site as shown with primers P1 + P2 (insertion of the resistance marker), P3 + P4 (disruption of TgME49_297720 at the gRNA cutting site), and P5 + P4 (insertion of the resistance cassette at the gRNA cutting site). Two clones, C2 and C3, were retained for subsequent investigations (Figure 1C).

**Figure 2:**
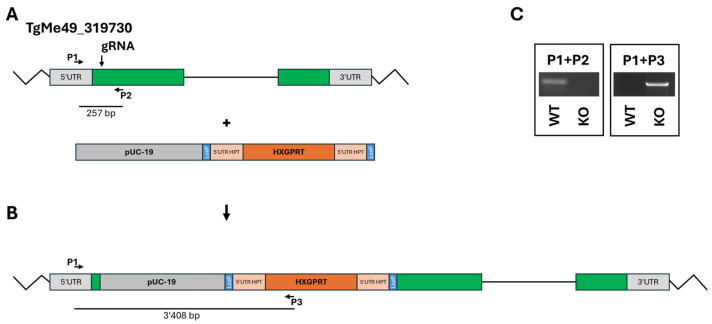
Disruption of the ORF TgME49_319730. (**A**), ORF plus insert; (**B**), final construct; (**C**), quality control PCRs. The localization of guide RNA and primers P1–P3 is indicated.

The KO of the ORF TgME49_319730 was achieved by inserting the resistance cassette at the 5′-end of the coding sequence (Figure 2A,B). One clone with correct insertion of the resistance cassette was retained for further studies (Figure 2C).

### 2.2. Growth Characteristics of the KO Clones

**Figure 3:**
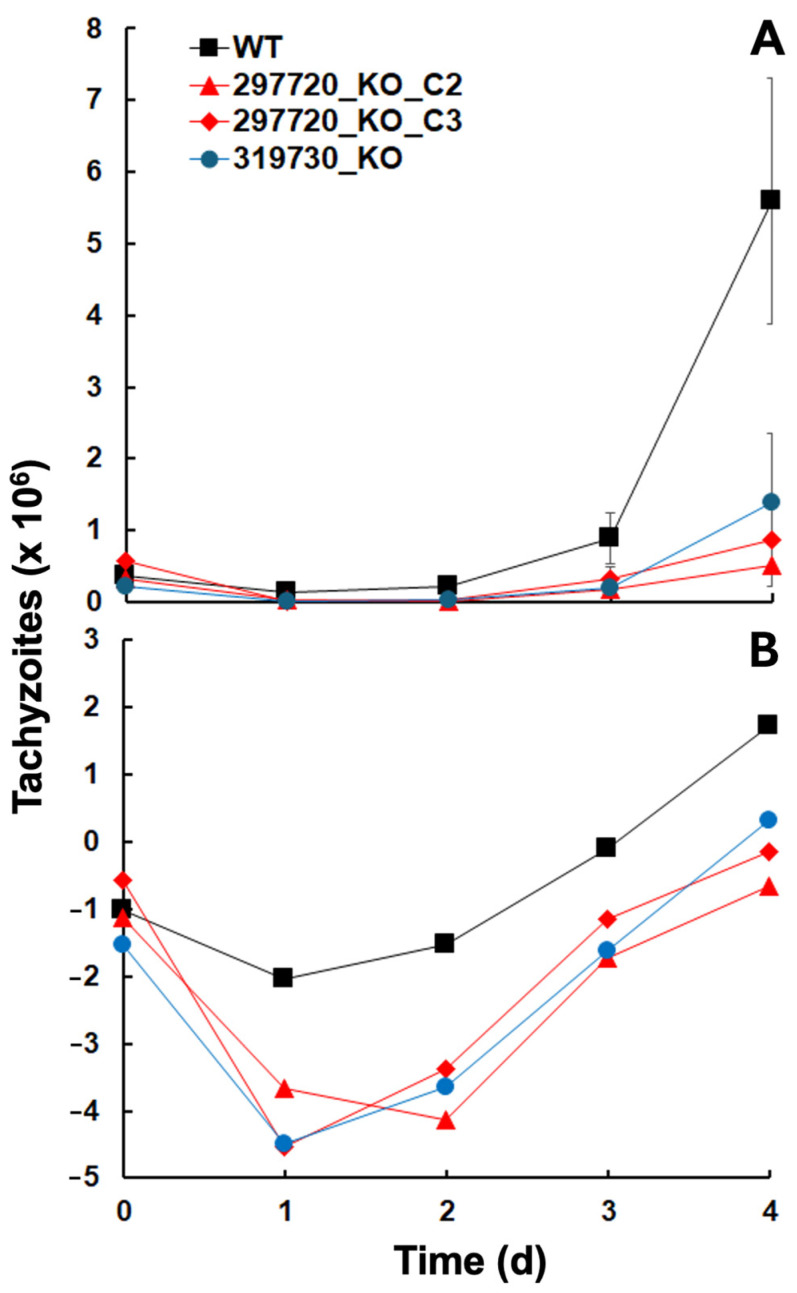
Growth of *T. gondii* ShSp1 wildtype (WT) and KO clone tachyzoites. (**A**), Untransformed data, (**B**), natural logarithms (ln) of A. Growth assays were performed in 24-well plates. Mean values ± standard deviations are given for three replicates.

KO clones of ORFs TgME49_297720 and 319730 could be generated, and deletions were not lethal, so the genes are not essential. However, when looking at the initial growth after inoculation of 10^5^ tachyzoites into confluent human foreskin fibroblast (HFF) cultures, it became evident that the corresponding wildtype resumed growth faster than all three KO clones (Figure 3A). A closer look at the logarithmically transformed data suggested that this was due to a decrease in the KO clone tachyzoite numbers during the first two days after inoculation (Figure 3B).

After 2 days, the growth of the KO clone tachyzoites resumed with similar growth rates as in wildtype tachyzoites (Table 1).

### 2.3. Differentially Expressed Proteins in Isolated Tachyzoites

In the first step, we analyzed the proteomes of isolated tachyzoites obtained from fully infected and lytic HFF cultures. Overall, 5367 non-redundant *T. gondii* proteins were identified in the analysis comparing two TGME_297720 knock-out strains (C2 and C3) with the corresponding wildtype *T. gondii* Sp1. Furthermore, 4185 proteins were identified in the analysis comparing a TGME_319730 knock-out strain with the corresponding wildtype strain (Table 2).

**Figure 4:**
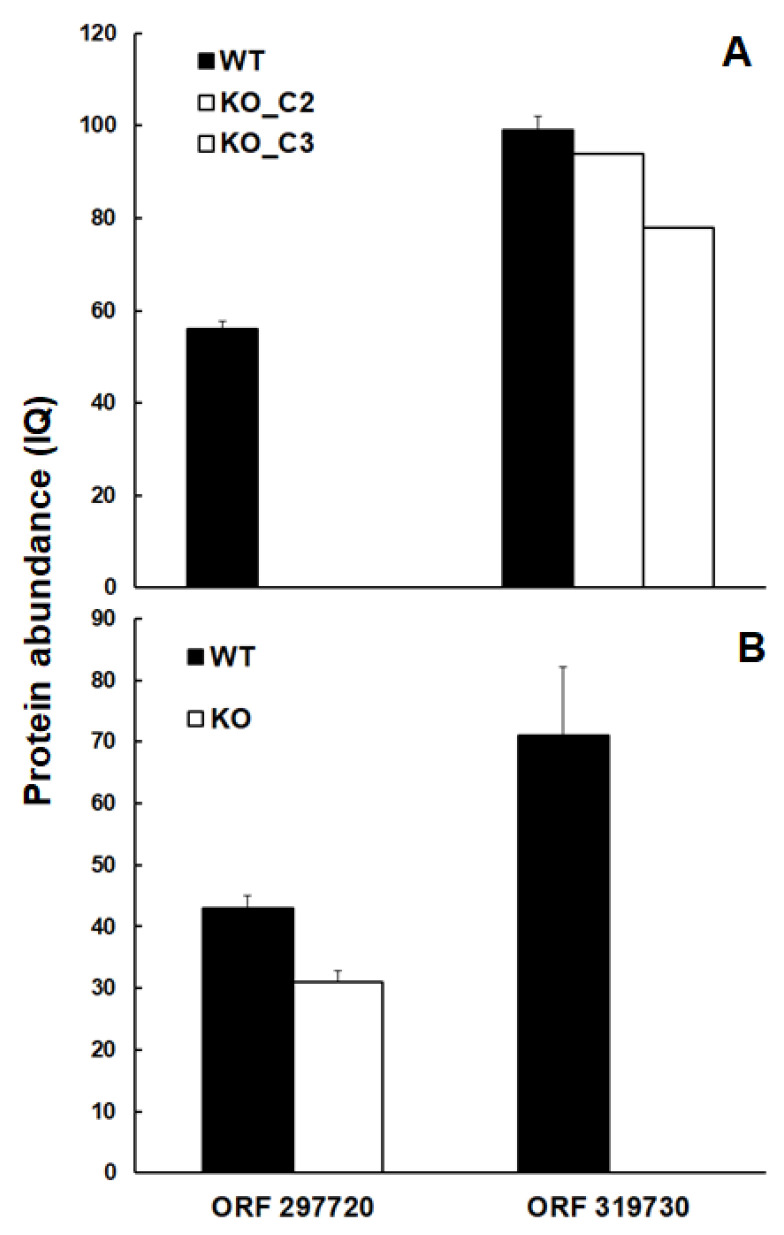
Abundances of intended KO proteins. The proteins encoded by ORFs TgME49_297720 and TgME49_319730 are present in wildtype tachyzoites (WT: black bars) but absent in TgME49_297720 (**A**), TgME49_319730, and (**B**) knock-out clones (white bars). Protein abundances measured as IQ (ion-based quantification) levels are given as mean values ± standard deviation for three independent replicates.

As shown in Figure 4, the trehalose-6-phosphate phosphatase homolog encoded by TGME49_297720 was well detectable in wildtype tachyzoites at comparative abundance levels in both experimental set-ups but was absent in the intended KO clones (Figure 4A).

The same was true for the protein encoded by TGME49_319730, which was well detectable in TGME49_297720 KO clones, but absent—as intended—in the TGME49_319730 KO clone (Figure 4B).

As expected, the proteins encoded by both ORFs subjected to KOs were absent in the respective clones. Considering the heterogeneity of the samples due to the KO, we have adapted the parameters usually applied to differential expression (DE) analysis by adding some extra filters to our scripts (Q values and PEP ≤ 0.01 at all levels and removal of low-intensity fragments). Only proteins with statistically significant differences by both ion-based quantification (IQ) and Top3 algorithms, calculated by the sum of the three most intense peptides coming from the same protein, were regarded as differentials.

**Figure 5:**
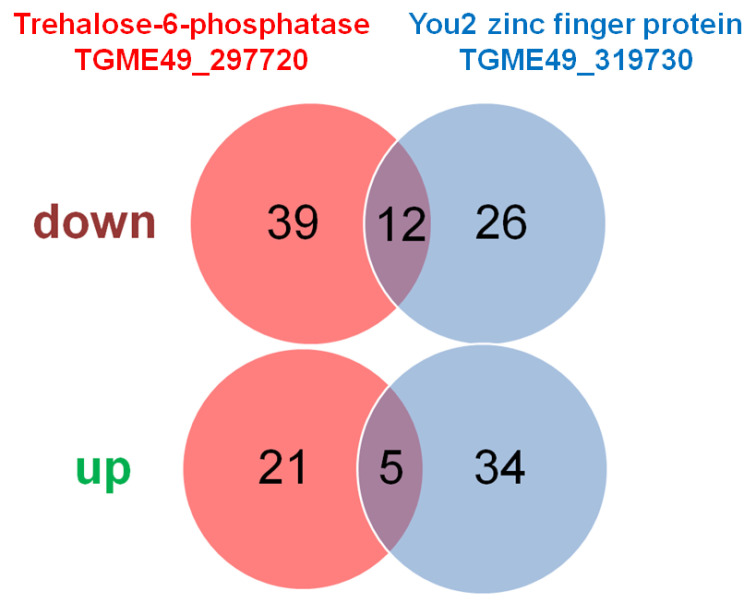
Overview of differentially expressed proteins in KO versus wildtype tachyzoites. The complete list of DE proteins is presented in Supplemental Table S3 (TgME49_297720 KO) and Table S4 (TgME49_319730 KO).

Overall, 77 DE proteins were identified in tachyzoites of both TGME49_297720 KO clones vs. wildtype tachyzoites, with 51 with significantly lower abundance levels in the KO clones, and 26 with higher abundance levels in the KO clones. The comparison of TGME49_319730 KO with wildtype tachyzoites yielded 77 DE proteins. A total of 38 DE proteins had lower expression levels in KO than in wildtype tachyzoites, while 39 DE proteins had higher expression levels in KO than in wildtype tachyzoites. Twelve proteins were commonly downregulated in both KO vs. WT datasets, while five were commonly upregulated (Figure 5). The complete subsets of DE proteins in the TGME_297720 KO clones are presented in Supplemental Table S3, and the DE proteins of the TGME49_319730 KO clone is given in Supplemental Table S4.

At first, we investigated DE proteins common to both KO experiments. Within the seventeen common DE proteins, the protein family with the highest number of common DE proteins were SAG-related sequence family proteins with four differentials (one down, three up) in total, followed by four hypothetical proteins. Interestingly, the subset of common downregulated proteins contained the typical bradyzoite markers lactate dehydrogenase LDH2, and the SAG-related sequence family protein SRS35A (Table 3).

In the next step, we had a closer look at the DE proteins specific to the TGME49_297720 KO tachyzoites. We divided the 60 specific differentials (see Table S3) according to protein families and functions. Besides eighteen hypothetical proteins not annotated in any of the *T. gondii* strains in ToxoDB, the most abundant subset is constituted by nine SAG proteins followed by eight proteins involved in intermediary metabolism or metabolite transport (Table 4).

Of particular interest within this subset are the eight proteins involved in metabolism. As shown in Table 5, amylo-alpha-1,6-glucosidase (TGME49_226910), the key enzyme of starch degradation was downregulated in the KO tachyzoites, as well as a key enzyme of the pentose-phosphate shunt, ribulose 5-phosphate isomerase (TGME49_239310).

As expected, the metabolic proteome pattern was different in tachyzoites of the KO strain of ORF TGME49_319730 as compared to both wildtype and TGME49_297720 KO tachyzoites (Table 6).

**Figure 6:**
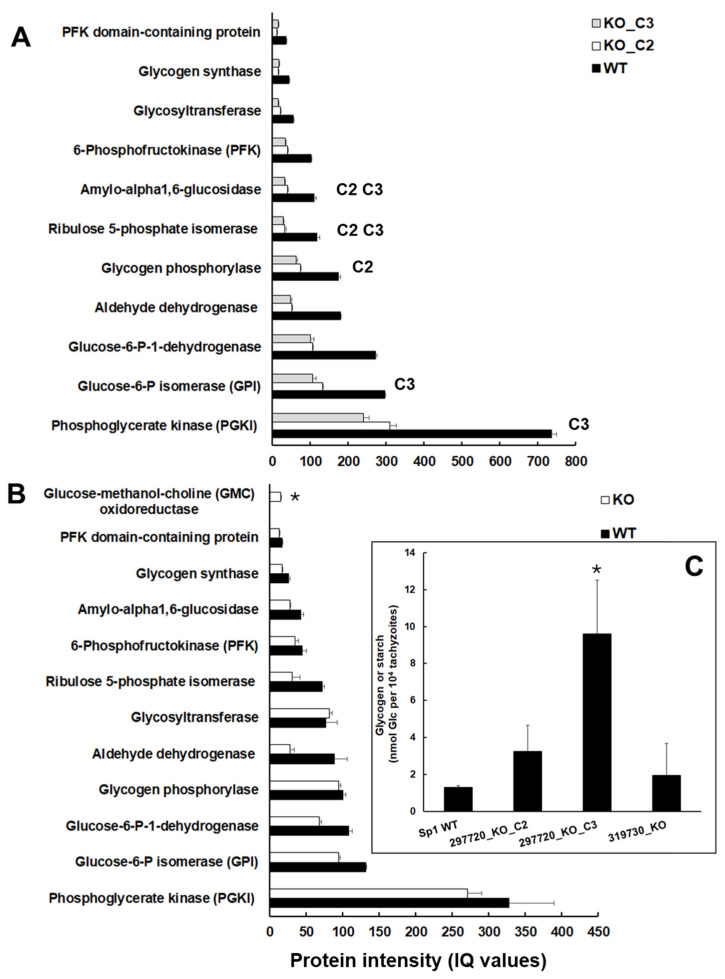
Expression of major glycogen/starch-degrading and glycolytic enzymes in *T. gondii* ShSp1 wildtype (WT) and KO lines. Protein intensities are indicated as ion-based quantification (IQ) values. (**A**), TGME49_297720 KO clones and wildtype. Superscribed letters indicate clones with significant DE. (**B**), TGME49_319730 KO and wildtype. Asterisk indicates significant DE. (**C**), Glycogen/starch content in the different clones. Asterisk indicates *p* < 0.05 as compared to wildtype. Mean values ± SD of three independent replicates are given.

These findings prompted us to investigate the protein intensities of glycogen/starch degrading and major glycolytic enzymes in the KO and wildtype tachyzoites in more detail. Moreover, we analyzed the glycogen/starch content in isolated tachyzoites of wildtype and KO tachyzoites. In TGME49_297720 clone tachyzoites, all enzymes were expressed at lower levels than in wildtype tachyzoites. As listed above, the levels of amyloglycosidase and ribulose-5-phosphate isomerase were significantly lower in both clones C2 and C3. Moreover, the levels of glycogen phosphorylase (TGME49_310670) were significantly lower in clone C2 only; the levels of glucose-6-phosphate isomerase (TGME49_283780) and phosphoglycerate kinase (TGME49_318230), thus of two glycolytic enzymes downstream of glycogen/starch degradation, were lower in clone C3 only (Figure 6A).

Conversely, despite tendentially lower levels, none of these enzymes had significantly lower levels in TGME49_319730 KO tachyzoites. Here, the only enzyme related to glucose metabolism with significant DE compared to wildtype was glucose-methanol-choline oxidoreductase with higher levels (Figure 6B). Consequently, it was not surprising to find higher glycogen/starch contents in clones C2 and, in particular, C3 but not in TGME49_319730 KO tachyzoites (Figure 6C).

### 2.4. Differentially Expressed Proteins in Infected Host Cells

In a next step, we analyzed host and parasite proteomes in infected HFF host cells. To do this, we infected confluent HFF layers and harvested the infected cells before they started to lyse. Overall, we identified 156,329 unique peptides matching 2178 *T. gondii* and 7755 *H. sapiens* proteins (Table 7 and Table S5).

**Figure 7:**
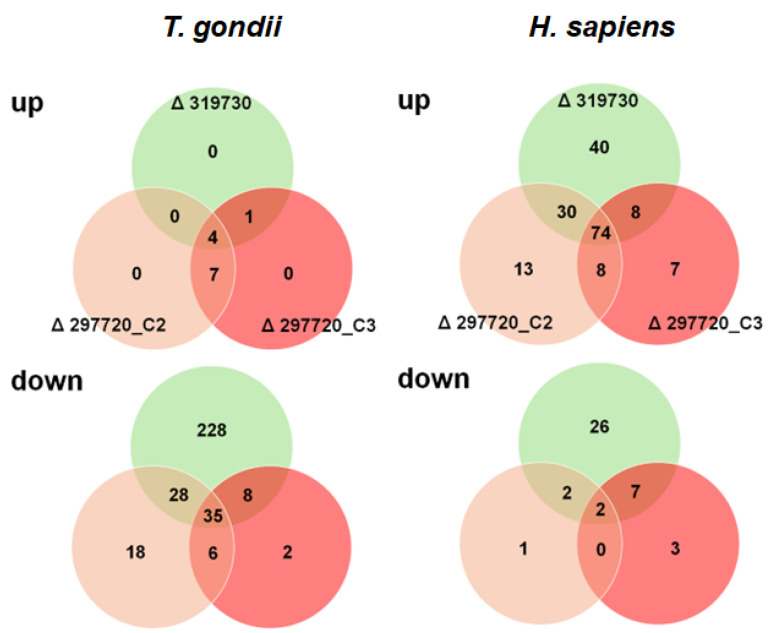
Overview of differentially expressed proteins in HFF-infected with KO versus wildtype tachyzoites. The complete list of DE proteins is presented in Supplemental Table S6 (*T. gondii* proteins) and S7 (*H. sapiens* proteins).

With respect to *T. gondii* proteins, only 12 DE proteins with significantly higher levels in cells infected with KO clones vs. cells infected with wildtype tachyzoites were detected. Interestingly, the number of *T. gondii* proteins with lower levels in KO clone vs. wildtype infected cells was one order of magnitude higher, namely 315 in total. The comparison of TGME49_319730 KO with wildtype tachyzoites alone yielded 228 DE proteins. Conversely, in cells infected with TGME49_297720 KO clones, only 26 DE proteins with lower levels in these clones could be identified (Figure 7; Table S6).

Comparing the *T. gondii* DE proteins identified in isolated tachyzoites at a late stage of infection and in infected host cells at an early stage of infection, we identified twenty-seven common DE proteins (twenty upregulated and seven downregulated). Both intended KOs, namely TGME49_297720 and TGME49_319730, were within the subset of downregulated proteins, and six additional proteins were downregulated in common, including the two typical bradyzoite markers SRS35A and LDH2, and two were upregulated in common, which were the GRA proteins 80 and 82. Whereas only six common DE proteins, two downregulated and four upregulated (including three SRS proteins), were identified in the TGME49_297720_KO clones, while twelve downregulated and one upregulated protein were identified in isolated TGME49_319730_KO tachyzoites, as well as in host cells infected with this strain (Table 8).

In host cells infected with both TGME49_297720_KO strain clones, only thirteen DE proteins were identified, whereby there were seven with higher abundance, and six with lower abundance (Table 9).

**Figure 8:**
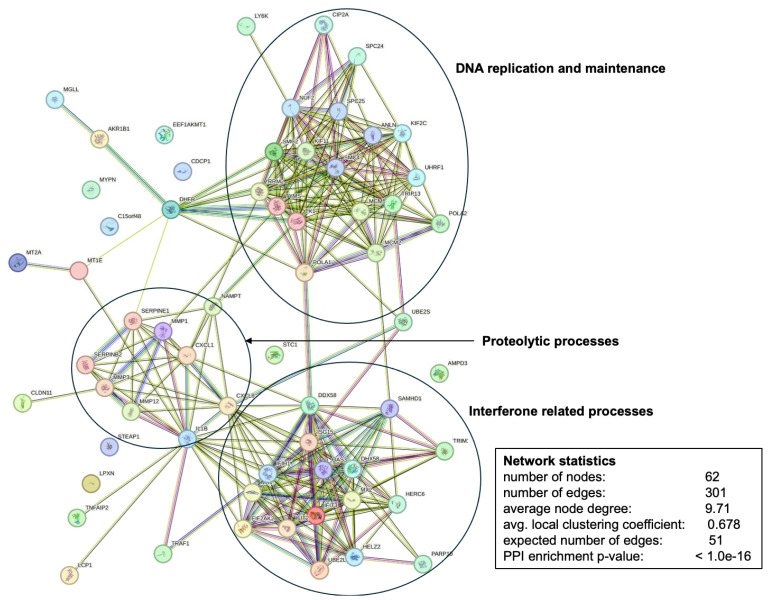
Interaction network of host proteins with significantly higher levels in *T. gondii* KO strain-infected vs. wildtype-infected cells. The PPI enrichment *p*-value is given as < 1.0e^−^16 instead of 10^−16^.

Our major interest in this study were the pleotropic effects of the KO clones vs. wildtype on host cell proteomes. In fact, we could identify 218 host cell proteins with DE in cells infected with KO vs. wildtype trophozoites, whereby 38 had lower expression levels in cells infected with KO than in cells infected with wildtype tachyzoites, while 180 had higher expression levels in cells infected with KO than in cells infected with wildtype tachyzoites (Figure 7). Of major interest for the present study were the 74 proteins commonly upregulated in cells infected with KO as compared to wildtype tachyzoites. A network analysis revealed that the majority of these proteins were grouped into three clusters with functions related to DNA replication and maintenance, as well as interferon-related and proteolytic processes (Figure 8).

The complete list of host cell DE proteins is presented in Supplemental Table S7.

**Figure 9:**
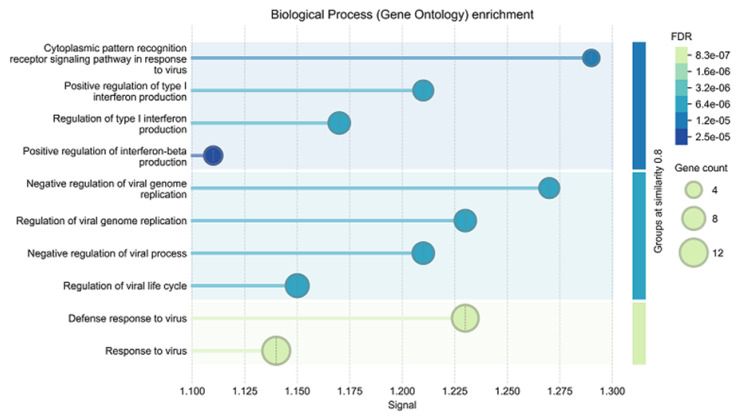
Biological processes enriched in host cells in *T. gondii* KO strain-infected vs. wildtype-infected cells. For better legibility, the false discovery rates (FDR) are given as e^−n^ instead of 10^−n^.

A gene ontology enrichment analysis of this network revealed a significant enrichment of biological processes related to antiviral responses within this network (Figure 9).

The complete list of host cell DE proteins is presented in Supplemental Table S7.

A second interaction network analysis of the 26 specific host cell proteins with higher intensities in cells infected with the TGME49_319730 KO strain as compared to cells infected with wildtype tachyzoites gave a different interaction network with a clear process enrichment in DNA replication and repair as shown in Supplemental Figure S1. In host cells infected with both TGME49_297720 KO strain clones, only eight DE host proteins (all upregulated) could be identified (Table S7).

## 3. Discussion

When performing knock-out (KO) or knock-in studies, the fundamental paradigm consists of attributing the observed effects in the target organism to the presence or absence of the gene of interest. This approach is valid only if there are no pleiotropic effects obliterating or masking the effects of interest. Moreover, in the case of intracellular organisms such as *T. gondii* (or other apicomplexans), the effects on host cells should also be considered. In the present study, we show that targeted gene knock-outs in *T. gondii* can lead to broader consequences than the simple loss of the gene of interest. Specifically, we observed significant shifts in the expression of both parasite and host proteomes, including sets of differentially expressed proteins that were shared across independent knock-outs.

Thus, before analyzing specific effects of the respective KOs, we examine common effects of both KOs on the tachyzoite proteomes. Concerning common downregulated proteins at early stages (i.e., within host cells) and at late stages (i.e., in isolated tachyzoites) of infection in KO vs. wildtype parasites, it is striking that the six proteins identified are upregulated in bradyzoite vs. tachyzoites (ToxoDB, accessed August 2025 and [12]). This suggests that the selection procedure following the transformation eliminates bradyzoites persisting in the untransformed wildtype from the cell population.

Only two proteins, namely GRA80 and GRA82, are upregulated in all KO vs. wildtype strains in intracellular as well as isolated tachyzoites. Both proteins are acidic and have signal peptides. Moreover, GRA80 has transmembrane domains (ToxoDB, accessed August 2025). GRA82 can be phosphorylated [13]. Both proteins are regarded as merozoite and thus cat-specific sexual stage markers were detected in infected cat intestinal tissues, and were also expressed at enhanced levels upon KOs of two transcription factors [14] and a F-box protein [15], which are in turn regarded as triggers for sexual development. Since our KO genes were different from the genes tagged in these studies, our results suggest that differential upregulation of these proteins could be caused by a pleiotropic effect due to the genetic manipulation procedure rather than specific effects due to the particular KOs.

Concerning specific effects on the KO of TGME49_297720 on tachyzoite proteomes, it is striking that—besides the intended KO—only five proteins are DE both in intracellular and purified tachyzoites, and three of them are upregulated SRS proteins. In purified KO clone tachyzoites—thus harvested at a later stage of infection—the DE proteome of this KO is larger. In particular, glycogen/starch-degrading and glycolytic enzymes are less abundant than in wildtype tachyzoites and in tachyzoites of the TGME49_319730_KO, and conversely, glycogen/starch levels are higher. This is insofar interesting as the gene product of TGME49_297720, a 1222 amino-acid protein annotated as trehalose-phosphatase, is homologous to genes having both trehalose-6-P-synthase/phosphatase domains found in insects, fungi, and plants with the highest identity to the protein CEL0385.1 of the marine algae *Vitrella brassicaformis* using the NCBI BLAST+ 2.17.0 server (https://blast.ncbi.nlm.nih.gov/Blast.cgi?PAGE=Proteins, E value 0, 4 August 2025). In plants, trehalose-6-phosphate (Tre-6-P) is involved in several signaling processes including carbohydrate metabolism [16]. Similar processes may occur in plant-related apicomplexans. We have, however, no functional evidence, that the gene product of ORF TGME49_297720 encodes a functional Tre-6-P synthase and/or phosphatase. In our hands, assays with the recombinant enzyme have failed. Substrates and products may be different from canonical Tre-6-P-synthases/phosphatases. Moreover, functional activity may depend on the phosphorylation status of the protein. In fact, phosphor–proteome analyses show that the TGME49_297720 protein has multiple phosphorylation sites [13,17], which may trigger the functional activity.

Concerning the ME49_319730_KO, the number of specific DE proteins is much higher in intracellular tachyzoites (228, all downregulated) than in isolated tachyzoites (60, 26 down-, 34 upregulated). The TGME49_319730 gene product is homologous to You2 C2C2 zinc finger proteins. In mammalians, these proteins are characterized as RNA-binding proteins controlling the expression of hundreds of target RNAs [18]. If TGME49_319730 has the same function, this could explain why its absence is correlated with lower expression levels of multiple proteins at early stages of infection. However, it is unclear how its alleged mitochondrial localization fits into this picture.

The most intriguing aspect of this study is the pleiotropic effects of the genetically manipulated strains on host cells. In host cells infected with all KO strains, 74 proteins have lower expression levels than in wildtype-infected cells. Enrichment analysis suggests that these proteins are involved in antiviral defense mechanisms. This may explain the transient depression in tachyzoite numbers of these KO strains observed early after infection. It is well known that after entering a host cell, *T. gondii* modulates its intracellular environment by modulating host cell gene expression and consequently host cell proteomes [19]. The modulating agents are proteins secreted via rhoptries [20] and dense granules [21]. In particular, GRA16 [22] and GRA24 [21] are discussed as major effectors since both reach the host nucleus and trigger host gene expression. In our dataset, GRA24 is upregulated in host cells infected with both TGME49_297720 KO clones. However, these clones have the smallest effect on host cells in terms of host DE protein numbers. In this context, it would be worthwhile investigating to which extent GRA80 and GRA82 contribute to the modulation of host cell gene expression.

Coming back to points (i.) to (iv.) raised in the introduction, we see that, while demonstration of points i. and ii. is standard, the proteomic part of the KO strain analysis—thus points iii. and iv.—are critical and therefore most often neglected. Unambiguous identification of the proteins of interest in the wildtype and absence of the protein in the KO strain (point iii.) needs a stringent statistical approach, as shown in the present study in the case of TGME_297720. Whole-cell proteome analysis (point iv.) shows that pleiotropic effects on both parasite and host cell gene expression have to be taken into account if genes of interest are investigated in genetically manipulated strains. Consequently, appropriate controls including analysis of unrelated KO strains are needed to narrow down the spectrum of potential functions of the gene of interest. Otherwise, potential interpretation errors are programmed. However, a limitation of this study is the low number of KO clones that are investigated, and further proteomic studies on other KO strains should be carried out to substantiate these findings, ideally by also incorporating direct functional assays. Finally, non-deterministic proteome changes at the cellular level as a consequence of manipulation of a single protein mimic the unpredictable changes seen in ecosystems as a consequence of modifications of single abiotic or biotic factors [23]. This is not surprising since it is well known that chaos or non-linear responses of systems to small perturbations is common to all scales of nature [24,25,26].

## 4. Materials and Methods

### 4.1. Chemicals

If not stated otherwise, all chemical used were purchased from Sigma (St. Louis, MO, USA). Cell culture media and fetal bovine serum (FBS) were from Bioswisstec (Schaffhausen, Switzerland).

### 4.2. In Vitro Culture and Parasite Maintenance

Human foreskin fibroblasts (HFF, ATCC, PCS-201-101TM) were maintained in Dubecco’s modified Eagle medium (DMEM) supplemented with 10% heat-inactivated and filter-sterilized calf serum (FCS), and 1% Antibiotic–Antimycotic (100×) as previously described [27]. TgShSp1 and corresponding knock-out tachyzoites were cultured as previously described [28].

### 4.3. Isolation of Parasites

Parasites were cultured in flasks until shortly before host cell lysis. To harvest single tachyzoites, cultures were scraped and mechanically disrupted by passing the suspension three times through a 25-gauge needle. Lysed cells were centrifuged at 800× *g* for 10 min and washed three times with PBS, using the same centrifugation conditions. Intracellular parasites were isolated using the same procedure, but without the lysis step.

### 4.4. Generation of the Knock-Out Strains

A TgShSp1 strain deficient in HPT was generated at the Complutense University of Madrid, Spain following the same methodology previously applied to other strains [29]. Selection of Δhpt-tachyzoites was achieved by culturing single clones in parallel with media containing mycophenolic acid (MPA) and xanthine (25 μg/mL each), and media containing 6-thioxanthine (177 μg/mL). Parasites that proliferated in 6-thioxanthine but not in MPA-xanthine media were chosen and verified via PCR.

The single-guide RNA (gRNA) targeting the genes TgME49_319730 and TgME49_297720 were designed using the ChopChop web tool (https://chopchop.cbu.uib.no/ (accessed on 9 September 2022)). The gRNA sequences were subsequently assessed for off-target effects using a Strawberry Perl script (https://strawberryperl.com/ (accessed on 9 September 2022)) in conjunction with the latest Toxoplasma genome type 2 data base (ToxoDB-59_TgondiiME49_Genome.fasta). Primer design was carried out with Primer3Plus (https://www.bioinformatics.nl/cgi-bin/primer3plus/primer3plus.cgi (accessed on 9 September 2022)), with verification of GC content, hairpin formation, and potential self- and hetero-dimerization using the OligoAnalyzer Tool (https://eu.idtdna.com/calc/analyzer (accessed on 9 September 2022)). Primer sequences were then compared against known sequences using the Nucleotide BLAST+ 2.17.0 tool (https://blast.ncbi.nlm.nih.gov (accessed on 9 September 2022)) The primer sequences are presented in Supplementary Table S8.

To disrupt the gene coding for either TgME49_319730 or TgME49_297720 in the TgShSp1Δhpt background strain, a gRNA sequence targeting the gene was cloned into the pU6 vector (Addgene plasmid # 52694) using the *BsaI*-specific sites.

For the TgME49_319730 knock-out, this plasmid was transfected alongside the linearized pUC-HPT plasmid, which contains the HPT selection cassette flanked by two loxP sites, facilitating a potential future excision of the HPT selection cassette via Cre recombinase [30]. The transfection mixture composed of a 5.6:1 vector-insert ratio, where 28 μg of the gRNA and 2.75 μg of the selection cassette were co-transfected. 5.1e7 parasites were electroporated using a Bio-Rad GenePulser Xcell (Biorad, Cressier, Switzerland) applying a 2 mm electroporation cuvette at the following settings: 1250 V voltage, 25 μF capacitance, and infinite resistance (ꚙ Ω).

For the TgME49_297720 knock-out, the plasmid containing the gRNA was transfected alongside the linearized pUC-HPT plasmid, containing homology parts at the 3′ and 5′ site of the 5′UTR and the 3′UTR of the TgME49_297720 gene, respectively. The plasmid for the HPT selection cassette containing the homology parts was created using the primers shown in Table S8 to obtain the individual parts and the plasmid was created using the HiFi DNA Assembly Kit and the NEBuilder online tool (New England Biolabs, Ipswich, MA, USA) according to manufacturer’s protocol. The plasmid was amplified using *E. coli* Top10. The transfection mixture composed of a 5:1 vector-insert ratio, where 35 μg of the gRNA and 4.55 μg of the selection cassette were co-transfected. 1.3 × 10^7^ parasites were electroporated using the Nucleoflector 2b with the program U-033.

### 4.5. Growth Analysis

Tachyzoite growth assays were performed in 24-well plates seeded with HFF monolayers. Confluent HFF monolayers were inoculated with 10′000 tachyzoites per well. The parasites were allowed to grow until 0, 24, 48, 72, and 96 h post-infection before being harvested. At these points, the infected cell layers as well as tachyzoites in the medium supernatant were pelleted by centrifugation for 5 min at 1000× *g*. Subsequently, DNA extraction of the pellets was performed using the NucleoSpin Rapid Lysis Kit (Machery-Nagel, Düren, Germany) according to the manufacturer’s instructions. The extracted samples were analyzed via diagnostic PCR targeting the 529 bp repetitive fragment of *T. gondii*, and the number of tachyzoites per well was quantified by comparison with a tachyzoite standard series [31].

### 4.6. Quantification of Glycogen/Starch

Glycogen/starch was quantified in isolated tachyzoites after extraction of soluble carbohydrates as described [32,33].

### 4.7. Proteomic Analysis of Isolated Tachyzoites

Protein extraction and processing for mass spectrometry was performed identically as described below for the infected host cells. The mass spectrometry analysis was performed as described earlier [34]. The data was searched and quantified with Spectronaut (Biognosys) version 19.9.250422.62635 in the hybrid direct data-independent acquisition (DIA)+ (deep) mode against the ToxoDB-55 [35] *T. gondii* ME49-annotated proteins (with added common contaminants). Factory settings were used, including precursor qvalue cutoff = 0.01, precursor PEP cutoff = 0.2, protein qvalue cutoff (Experiment) = 0.01, protein qvalue cutoff (Run) = 0.05, and protein PEP cutoff = 0.75. Further parameters were as follows: cleavage rule = trypsin/P with maximum two missed cleavages, Carbamidomethyl (C) as fixed modification, and Acetyl (Protein N-term) and Oxidation (M) as variable modifications (maximum 5). Single hit proteins were excluded.

### 4.8. Proteomic Analysis of Infected Host Cells

Cell pellets were lysed in 100 μL 8 M Urea/100 mM Tris-HCl pH8, containing proteases inhibitor cocktail (Complete EDTA free, Roche, Mannheim, Germany) using 6 bursts of 10 s by a probe sonicator. Proteins were then reduced with 10 mM dithiothreitol (DTT) for 30 min at 37 C, alkylated with 50 mM Iodoacetamide for 30 min at RT in the dark, and precipitated with 5 volumes of acetone for two hours at −20 °C. Proteins were sedimented by centrifugation for 10 min at 13,000 rpm and 4 °C, and the supernatant discarded; the pellet was air dried for 15 min. Proteins were reconstituted in 30 μL 8 M Urea/50 mM Tris-HCl pH8 and protein concentration was determined by Bradford assay. An aliquot corresponding to 10 μg protein was digested for 2 h at 37 °C with sequencing grade endoproteinase LysC (Promega, Madison, WI, USA) after dilution of urea to 4 M with 20 mM Tris/HCl pH 8.0, 2 mM calcium dichloride, followed by overnight at room temperature with sequencing grade trypsin (Promega) at a urea concentration of 1.6 M. Digests were acidified with TFA (1% end concentration) and 400 ng of the digests were analyzed on a nano-liquid chromatography tandem mass spectrometer system consisting of a Vanquish Neo ultra-performace liquid chromatography (UPLC) and an Orbitrap Astral (ThermoFisher Scientific, Bermen, Germany) by loading peptides onto a C18 trap-column (PepMap 100, 5 µm, 100 Å, 300 µm i.d. ×5 mm length, ThermoFisher) at 80 bar pressure using a solvent consisting of 0.05% trifluoroacetic acid (TFA) in a water/acetonitrile mixture (98:2). Peptides were then eluted in backflush mode onto a homemade C18 CSH Waters column (1.7 μm, 130 Å, 75 μm × 20 cm) using a 22 min gradient of 5% to 40% acetonitrile in water containing 0.1% formic acid, at a flow rate of 300 nL/min. Each sample was analyzed twice, first with the data-dependent acquisition (DDA) method acquiring full scan data in the orbitrap (resolution 240′000, 380–980 m/z, normalized AGC of 300%, maximum injection time of 5 ms) and fragment spectra of peptides with charge states 2–6 in the Astral analyzer (normalized HCD energy of 28%, AGC of 80%, maximum injection time of 5 ms, scan range 120–1800 m/z, precursor exclusion for 20 s). A data-independent acquisition (DIA) mode was applied with a full scan in the orbitrap every 0.6 s (resolution 240′000, 380–980 m/z, normalized AGC of 500%, maximum injection time of 5 ms) and 299 MS2 scans of 2 m/z isolation width, with a scan range of 150–2000 m/z, and normalized AGC of 500% with a maximum injection time of 3 ms, and normalized HCD energy of 28%.

The data was searched and quantified with Spectronaut (Biognosys) version 20.1.250624.92449 in the directDIA+ (deep) mode against the ToxoDB-68 TgondiiME49-annotated proteins [35], concatenated to the UniProt [36] human sequences (release 2025_01); common contaminants were also added. Search parameters were 10 ppm and 20 ppm MS1, respectively; MS2 mass tolerance, precursor with protein q value, and PEP cutoffs were set to 0.01. Further parameters were as follows: cleavage rule = trypsin with maximum 2 missed cleavages, Carbamidomethyl (C) as fixed modification, and Acetyl (Protein N-term) and Oxidation (M) as variable modifications (maximum 3). Single hit proteins were excluded.

### 4.9. Statistics

Protein groups not flagged as potential contaminants were retained for further analysis. Following Pham et al. [37], the distribution of fragment peak areas reported by Spectronaut was inspected and the fragments with the lowest intensities, or not used for quantification, were removed. A further filtering step, relevant for the isolated tachyzoites datasets, consisted of enforcing the precursor and protein q value and PEP cutoffs of 0.01. Based on this selection, a leading protein was chosen per protein group on the basis of best coverage. The ion-based quantification (IQ) [37] (open-sourced-based maxLFQ) was obtained with the R package iq (version 1.10.1) for each protein group after median normalization of the fragment intensities. A Top3 [38] implementation was also provided: peptide intensities were constructed from the sum of fragment intensities and normalized by variance stabilization [39]; a same set of Top3 peptides per protein were chosen for all samples based on the sum of intensity across samples, thereby preventing minority peptides from contributing to the intensity. Moreover, proteins with less than 2 chosen peptides in a sample were deemed as not detected in this sample.

Differential expression between two groups of replicates was calculated, provided that at minimum two identifications existed in at least one group of replicates. Missing values were imputed at protein group level for the IQ measures, and at the peptide level for the Top3 measure. If there was at most one non-zero value in the replicate group for a protein group, then the missing values were imputed by drawing random values from a Gaussian distribution of width 0.3 × sample standard deviation centered at the sample distribution mean minus 2.5 × sample standard deviation at protein level, respectively, with 2.8 × sample standard deviation at peptide level. Any remaining missing values were imputed by the Maximum Likelihood Estimation (MLE) method [40]. Differential expression tests were performed with the moderated *t*-test of the R limma package [41]. The Benjamini and Hochberg [42] method was then applied to correct for multiple testing. The criterion for statistically significant differential expression was that the largest accepted adjusted *p*-value reaches 0.05 asymptotically for large absolute values of the log2 fold change, and tends to 0 as the absolute value of the log2 fold change approaches 1 (with a curve parameter of 0.1x overall standard deviation) as described recently [43]. Proteins consistently significantly differentially expressed through 20 imputation cycles for both the IQ and the Top3 measures were flagged accordingly and retained as differentially expressed.

## Figures and Tables

**Table 1:** Logarithmic growth rates and doubling times of *T. gondii* ShSp1 wildtype and KO clones. Growth analyses were performed on the dataset presented in Figure 3.

Strain	Logarithmic Growth Rate (d^−1^)	Doubling Time (d)
*T. gondii* Sp1 wildtype	1.63	0.43
297720_KO_C2	1.73	0.40
297720_KO_C3	1.62	0.43
319730_KO	1.99	0.35

**Table 2:** Summary of protein quantification data of isolated tachyzoites. Tachyzoites of knock-out (KO) clones of two ORFs of *T. gondii* ME49 and of wildtype (WT) tachyzoites were subjected to whole-cell LC-MS/MS shotgun analysis and subsequent analysis of differential abundance as described in Materials and Methods. Three biological replicates were tested for each strain.

Parameter	TGME49_297720 KO vs. WT	TGME49_319730 KO vs. WT
Unique *T. gondii* peptides	113,213	60,863
Non-redundant *T. gondii* proteins	5367	4185
Datasets	Table S1	Table S2

**Table 3:** Common DE proteins in KO strains of TGME49_297720 and TGME49_319730. Tachyzoites of knock-out (KO) clones and of wildtype (WT) tachyzoites were subjected to whole-cell LC-MS/MS shotgun analysis and subsequent analysis of differential expression is described in Materials and Methods.

Common Downregulated Proteins		Common Upregulated Proteins	
GN	Description	GN	Description
TGME49_200250	Microneme protein MIC17A	TGME49_224770	SAG-related sequence SRS40D
TGME49_203720	Vitamin k epoxide reductase family protein	TGME49_273980	Hypothetical protein
TGME49_207210	Hypothetical protein	TGME49_277230	Hypothetical protein
TGME49_209985	cAMP-dependent protein kinase	TGME49_292260	SAG-related sequence SRS36B
TGME49_213480	Hypothetical protein	TGME49_320250	SAG-related sequence SRS15A
TGME49_243680	Dihydrodipicolinate reductase		
TGME49_280570	SAG-related sequence SRS35A		
TGME49_291040	Lactate dehydrogenase LDH2		
TGME49_308093	Rhoptry kinase family protein (incomplete catalytic triad)		
TGME49_309930	Melibiase subfamily protein GRA65		
TGME49_318880	Hypothetical protein		
TGME49_319090	IgA-specific serine endopeptidase		

**Table 4:** Overview of DE proteins specific to KO strains of TGME49_297720 and TGME49_319730. Tachyzoites of knock-out (KO) clones and of wildtype (WT) tachyzoites were subjected to whole-cell LC-MS/MS shotgun analysis and subsequent analysis of differential expression is described in Materials and Methods.

Protein Family or Function	TGME49_297720		TGME49_319730	
	KO Down	KO Up	KO Down	KO Up
Metabolism or transport	8	0	5	6
Signaling	3	1	0	3
Protein binding or modification	4	1	3	0
Nucleic acid binding or modification	6	3	6	3
BAG or MAG	1	0	1	0
GRA	0	1	1	2
MIC	3	0	1	0
ROP	2	0	0	1
SAG	4	5	2	6
Unknown	8	10	7	13
**Sum**	**39**	**21**	**26**	**34**

**Table 5:** Differentially expressed proteins involved in metabolism wildtype versus KO strains of TGME49_297720. Tachyzoites of two knock-out (KO) clones and of wildtype (WT) tachyzoites were subjected to whole-cell LC-MS/MS shotgun analysis and subsequent analysis of differential expression as described in Materials and Methods. All DE proteins listed here had lower expression levels in KO clones as compared to wildtype tachyzoites. The metabolic steps are annotated based on ToxoDB (www.toxodb.org) information (accessed on 4 August 2025).

GN	Description	Metabolic Step
TGME49_226910	Amylo-alpha-1,6-glucosidase	Starch degradation
TGME49_231920	Oxidoreductase, short chain dehydrogenase/reductase family protein	Unknown redox process
TGME49_238200	Alpha/beta hydrolase fold domain-containing protein	Unknown hydrolytic process
TGME49_239310	Ribulose 5-phosphate isomerase	Pentose phosphate shunt
TGME49_257750	Homocysteine s-methyltransferase domain-containing protein	Methionine biosynthesis
TGME49_297720	Trehalose-phosphatase (the intended KO)	Trehalose biosynthesis
TGME49_313050	Oxidoreductase, short chain dehydrogenase/reductase family protein	Unknown redox process
TGME49_320630	Phosphotransferase enzyme family protein	Activation of alcohol group

**Table 6:** Differentially expressed proteins involved in metabolism in wildtype versus KO strain of TGME49_319730. Tachyzoites of knock-out (KO) and of wildtype (WT) tachyzoites were subjected to whole-cell LC-MS/MS shotgun analysis and subsequent analysis of differential expression is described in Materials and Methods. The metabolic steps are annotated based on ToxoDB (www.toxodb.org) information (accessed on 4 August 2025).

GN	Description	Metabolic Step
*Downregulated in KO*		
TGME49_215490	Novel putative transporter NPT1	Transmembrane transport
TGME49_222160	Aldehyde dehydrogenase	Cytosolic redox process
TGME49_266640	Acetyl-coenzyme A synthetase 2, putative	Acetyl-CoA biosynthesis
TGME49_268860	Enolase 1	Glycolysis in bradyzoites
TGME49_301210	NAD(P) transhydrogenase subunit beta, putative	NADH-NADPH interconversion
*Upregulated in KO*		
TGME49_219230	AMP-binding enzyme domain-containing protein	Lipid metabolism
TGME49_227100	Glutaredoxin 5	Fe-S-cluster biosynthesis
TGME49_277240	NTPase I	Nucleotide hydrolysis
TGME49_294640	Ribonucleoside-diphosphate reductase large chain	dNTP biosynthesis
TGME49_313950	Glucose-methanol-choline (GMC) oxidoreductase	Various redox processes
TGME49_321570	beta-hydroxyacyl-acyl carrier protein dehydratase (FABZ)	Lipid metabolism

**Table 7:** Summary of protein quantification data of infected host cells. Tachyzoites of knock-out (KO) clones of two ORFs of T. gondii ME49 and of wildtype (WT) tachyzoites were subjected to whole-cell LC-MS/MS shotgun analysis and subsequent analysis of differential expression is described in Materials and Methods. Three biological replicates were tested for each strain. The completed dataset is given in Supplemental Table S5.

	*T. gondii*	*H. sapiens*
Unique peptides	156,329	
Non-redundant proteins	2178	7755
Data bases	ToxoDB	Uniprot

**Table 8:** Common DE *T. gondii* proteins in HFF host cells and in isolated tachyzoites. Host cells infected with knock-out (KO) or with *T. gondii* Sp1 wildtype (WT) tachyzoites were subjected to whole-cell LC-MS/MS shotgun analysis and subsequent analysis of differential expression is described in Materials and Methods. The DE proteins were compared with the DE proteins listed in Tables S3 and S4.

GN	Description	KO Strain
*Downregulated in KO*		
TGME49_203720	Vitamin k epoxide reductase family protein	All KO strains
TGME49_207210	Hypothetical protein	All KO strains
TGME49_209985	Rhoptry protein ROP42	All KO strains
TGME49_280570	SAG-related sequence SRS35A	All KO strains
TGME49_291040	Lactate dehydrogenase LDH2	All KO strains
TGME49_309930	Dense granule protein GRA56	All KO strains
TGME49_258230	Rhoptry kinase family protein ROP20	TGME49_297720 KO clones C2 and C3
TGME49_297720	Trehalose-6-P phosphatase (the intended KO)	TGME49_297720 KO clones C2 and C3
TGME49_200250	Microneme protein MIC17A	TGME49_319730 KO clone
TGME49_202020	DnAK-TPR	TGME49_319730 KO clone
TGME49_207160	SAG-related sequence SRS49D	TGME49_319730 KO clone
TGME49_207210	Hypothetical protein	TGME49_319730 KO clone
TGME49_209755	Cyst matrix protein MAG2	TGME49_319730 KO clone
TGME49_216140	Tetratricopeptide repeat-containing protein ANK1	TGME49_319730 KO clone
TGME49_222160	Aldehyde dehydrogenase	TGME49_319730 KO clone
TGME49_260600	mRNA-binding protein PUF1	TGME49_319730 KO clone
TGME49_264660	SAG-related sequence SRS44	TGME49_319730 KO clone
TGME49_308093	rhoptry kinase family protein (incomplete catalytic triad)	TGME49_319730 KO clone
TGME49_319560	Microneme protein MIC3	TGME49_319730 KO clone
TGME49_319730	You2 zinc finger protein (the intended KO)	TGME49_319730 KO clone
*Upregulated in KO*		
TGME49_273980	Dense granule protein GRA80	All KO clones
TGME49_277230	Dense granule protein GRA82	All KO clones
TGME49_224760	SAG-related sequence SRS40E	TGME49_297720 KO clones C2 and C3
TGME49_239090	SAG-related sequence SRS23	TGME49_297720 KO clones C2 and C3
TGME49_278370	*Toxoplasma gondii* family A protein	TGME49_297720 KO clones C2 and C3
TGME49_292260	SAG-related sequence SRS36B	TGME49_297720 KO clones C2 and C3
TGME49_322010	Myosin-light-chain kinase	TGME49_319730 KO clone

**Table 9:** *T. gondii* DE proteins in HFF host cells infected with both C2 and C3 TGME49_297720_KO strain clones. Host cells infected with two knock-out (KO) or with *T. gondii* Sp1 wildtype (WT) tachyzoites were subjected to whole-cell LC-MS/MS shotgun analysis and subsequent analysis of differential expression is described in Materials and Methods. See Table S6 for more information. Remarks according to https://toxodb.org (accessed on 4 August 2025).

GN	Description	Remarks
*Downregulated in KO*		
TGME49_207930	Phosphatidylethanolamine-binding protein	Dense granule protein, expression in bradyzoites and oocysts
TGME49_232780	Basal complex component BCC1	Phosphorylated, also present in oocysts
TGME49_258230	Rhoptry kinase family protein ROP20	DE also in isolated tachyzoites
TGME49_268860	Enolase 1	Bradyzoite marker
TGME49_297720	Trehalose-phosphatase	Intended KO
TGME49_313780	Hypothetical protein	Present in tachyzoite conoid proteome
*Upregulated in KO*		
TGME49_224760	SAG-related sequence SRS40E	DE also in isolated tachyzoites
TGME49_230180	Dense granule protein GRA24	Virulence marker
TGME49_239090	SAG-related sequence SRS23	DE also in isolated tachyzoites
TGME49_265870	Pantoate-beta-alanine ligase	Nucleus located, phosphorylated
TGME49_278370	*Toxoplasma gondii* family A protein	DE also in isolated tachyzoites
TGME49_292260	SAG-related sequence SRS36B	DE also in isolated tachyzoites
TGME49_308950	Histidine acid phosphatase superfamily protein	Dense granule protein

## Data Availability

The original proteome data presented in this study are included in the article/Supplementary Materials. Further inquiries can be directed to the corresponding author.

## Supplementary Materials

The following supporting information can be downloaded at https://www.mdpi.com/article/10.3390/ijms262110433/s1.
