# Supplementary material for: Pleiotropic Effects on Tachyzoite and Host Cell Proteomes in Knock-Out Clones of the Open Reading Frames 297720 and 319730 Constitutively Expressed in T. gondii ShSp1 Tachyzoites
Source: Int J Mol Sci. 2025 Oct 27;26(21):10433. doi: 10.3390/ijms262110433 (PMC12607534; doi:10.3390/ijms262110433)
Supplement: Supplementary file 1 [file ijms-26-10433-s001.zip › FigureS1.pptx]

## Slide 1
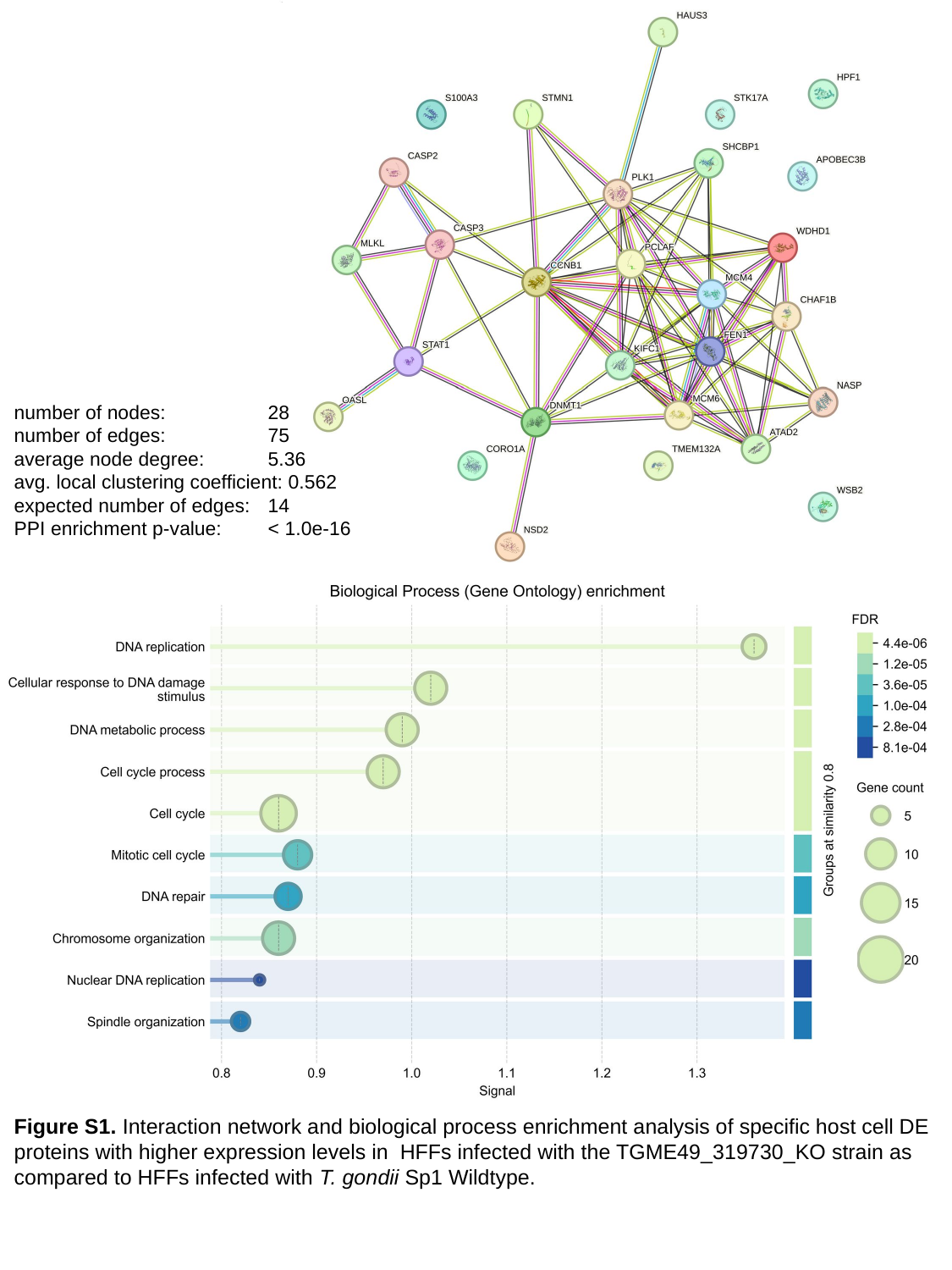

number of nodes:	28
number of edges:	75
average node degree:	5.36
avg. local clustering coefficient: 0.562
expected number of edges:	14
PPI enrichment p-value:	< 1.0e-16
Figure S1. Interaction network and biological process enrichment analysis of specific host cell DE proteins with higher expression levels in HFFs infected with the TGME49_319730_KO strain as compared to HFFs infected with T. gondii Sp1 Wildtype.
