# Supplementary material for: Pleiotropic Effects on Tachyzoite and Host Cell Proteomes in Knock-Out Clones of the Open Reading Frames 297720 and 319730 Constitutively Expressed in T. gondii ShSp1 Tachyzoites
Source: Int J Mol Sci. 2025 Oct 27;26(21):10433. doi: 10.3390/ijms262110433 (PMC12607534; doi:10.3390/ijms262110433)
Supplement: Supplementary file 1 [file ijms-26-10433-s001.zip › TableS8.pdf]

Table S8. Names and sequences of primers used for generation and validation of the knockout strains. Nucleotides highlighted in italics are part of the *BsaI* cloning site of the pU6 vector (Addgene plasmid #52694), and nucleotides in bold are the actual sgDNA sequence. The underlined bases have been added to increase specificity.

Name	Sequence
TGME49_297720 KO	
gRNA_TgME49_297720_fwd	*AAGTT*G**AATCATCAACATGCCTGTGG***G*
gRNA_TgME49_297720_rev	*AAAAC***CCACAGGCATGTTGATGATT**C*A*
TgME49_297720_P1	CCAGTGACGACGAGTGTTGA
TgME49_297720_P2	CAACTCCTCGCCGAAGTAAG
TgME49_297720_P3	TCTCGACTTGTCCATCCCCT
TgME49_297720_P4	GAAGTGGGCGTTGTTTACCG
TgME49_297720_P5	ACAGTCTCACCTCGCCTTGT
TGME49_319730 KO	
gRNA_TgME49_319730_fwd	*AAGTT*G**CGGTACCCTTCGCTGGAGGA***G*
gRNA_TgME49_319730_rev	*AAAAC***TCCTCCAGCGAAGGGTACCG**C*A*
TgME49_319730_P1	TTTTCACCTTCGGTCTCTCG
TgME49_319730_P2	CGTTCGAGTGCTGTGTGTCT
TgME49_319730_P3	CAACTCCTCGCCGAAGTAAG
